# Working alliance, interpersonal trust and perceived coercion in mental health review hearings

**DOI:** 10.1186/1752-4458-5-29

**Published:** 2011-11-10

**Authors:** Vidis Donnelly, Aideen Lynch, Damian Mohan, Harry G Kennedy

**Affiliations:** 1 National Forensic Mental Health Service, Central Mental Hospital, Dundrum, Dublin 14, Ireland; 2 Department of Psychiatry, Trinity College, Dublin, Ireland

## Abstract

**Background:**

There is some evidence that when mental health commitment hearings are held in accordance with therapeutic jurisprudence principles they are perceived as less coercive, and more just in their procedures leading to improved treatment adherence and fewer hospital readmissions. This suggests an effect of the hearing on therapeutic relationships. We compared working alliance and interpersonal trust in clinicians and forensic patients, whose continued detentions were reviewed by two different legal review bodies according to their legal category.

**Methods:**

The hearings were rated as positive or negative by patients and treating psychiatrists using the MacArthur scales for perceived coercion, perceived procedural justice (legal and medical) and for the impact of the hearing. We rated Global assessment of Function (GAF), Positive and Negative Symptom Scale (PANSS), Working Alliance Inventory (WAI) and Interpersonal Trust in Physician (ITP) scales six months before the hearing and repeated the WAI and ITP two weeks before and two weeks after the hearing, for 75 of 83 patients in a forensic medium and high secure hospital.

**Results:**

Psychiatrists agreed with patients regarding the rating of hearings. Patients rated civil hearings (MHTs) more negatively than hearings under insanity legislation (MHRBs). Those reviewed by MHTs had lower scores for WAI and ITP. However, post-hearing WAI and ITP scores were not different from baseline and pre-hearing scores. Using the receiver operating characteristic, baseline WAI and ITP scores predicted how patients would rate the hearings, as did baseline GAF and PANSS scores.

**Conclusions:**

There was no evidence that positively perceived hearings improved WAI or ITP, but some evidence showed that negatively perceived hearings worsened them. Concentrating on functional recovery and symptom remission remains the best strategy for improved therapeutic relationships.

## Background

It has been hypothesised that many aspects of legal process can be regarded as therapeutic jurisprudence, the study of the law as a therapeutic agent, in particular the law's impact on emotional life and on psychological well-being [1]. If the law can be used as a therapeutic agent, then therapeutic relationships and outcomes should be considered in this context. There is evidence that a fair and transparent legal process may have beneficial effects on clinical outcomes, and there is evidence to support this in relation to mental health hearings at the point of committal [2,3]. When post-committal hearings are allowed to become adversarial rather than inquisitorial, they may damage the therapeutic relationship between the patient and the treating psychiatrist, who is called upon in the hearings to justify continued detention [4]. This might in part be due to the legal language and styles of debate fostered by adversarial legal procedures.

There is a general lack of knowledge about tribunal procedures among patients [5]. In high security hospitals in England & Wales more than 90% of hearings led to no changes in the patients' legal status after the Tribunals [6]. In England and Wales there was growing concern that the Mental Health Review Tribunal (MHRT) system was becoming increasingly legalistic. Ferenz and McGuire [7] investigated the experiences of patients and tribunal members. They concluded that each group had different views. Patients were significantly less likely to agree that the tribunal was fair.

In Ireland, Mental Health Tribunals for the review of detention under the Mental Health Act (2001) were introduced for the first time in November 2006. Mental Health Review Boards to review detention under the Criminal Law (Insanity) Act (2006) commenced in January 2007. These were required to comply with the European Convention on Human Rights. We set out to examine the impact of these hearings on the patients' perception of working alliance and interpersonal trust in clinicians.

We hypothesised that when hearings are perceived negatively, there would be a decline in measured working alliance or interpersonal trust, or both. In this jurisdiction, there are two different bodies reviewing the detention of patients in a forensic hospital, according to whether they are detained under civil or criminal mental health legislation. Therefore, we had a unique opportunity to compare the two. We hypothesised that the subjective appraisal of working alliance and interpersonal trust, like the subjective appraisal of perceived coercion in mental health hearings, might be confounded by measures of mental state and global function.

## Methods

### Study design

In order to measure the effects of mental health review hearings on therapeutic relationships, we asked patients to complete two well validated self-report measures, the Working Alliance Inventory (WAI) [8] and the Interpersonal Trust in Physician scale (ITP) [9]. We have previously described our modification of these instruments and validation of their use in patients with severe mental illnesses detained in a forensic hospital [10]. The modified WAI (with the permission of the author) is given in additional file 1 along with the modified ITP.

**Figure 1:**
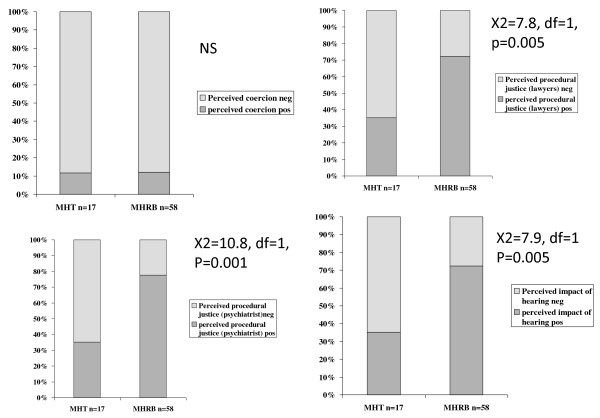
**Patients' appraisals of two types of hearings**. Patients' ratings of hearings as coercive, procedural justice regarding the role of legal chair and their own solicitor and procedural justice regarding their psychiatrist, and the impact of the hearing.

All patients, their consultant psychiatrists and their primary nurses were asked to complete the WAI and ITP in the month of April 2008 and again on average six months later, two weeks prior to their next Mental Health Tribunal (MHT, under the civil Mental Health Act 2001) or Mental Health (Criminal Law) Review Board (MHRB, under the Criminal Law (Insanity) Act 2006) [11]. Immediately after the hearings, patients and their treating consultant psychiatrists were asked to complete assessments of perceived coercion and perceived procedural justice using the MacArthur instruments for measuring perceived coercion, perceived procedural justice and the impact of the hearing [2,3] to dichotomise hearings into positive or negative. The content of the ratings of perceived coercion, perceived procedural justice and impact of the hearing is given in additional file 2. Two weeks after the hearing, patients and clinicians were again asked to complete the WAI and ITP. Figure 1 gives a schematic representation of the protocol.

The GAF [12] and PANSS [13] were measured at baseline so that these could be considered as possible confounders. These were measured by assistant psychologists who were blind to the ratings of WAI and ITP.

### Setting

In Ireland there is only one therapeutically secure forensic hospital, the Central Mental Hospital, where patients can be detained under the Criminal Law (Insanity) Act 2006. The Central Mental Hospital can also accept patients detained under civil mental health legislation, the Mental Health Act 2001, if their transfer from a local hospital is approved by a mental health tribunal. Patients detained under the Criminal Law (Insanity) Act 2006 are reviewed every six months by the Mental Health Review Board (MHRB) established under the Act of 2006. Patients detained under the Act of 2001 are reviewed at legally fixed intervals by a Mental Health Tribunal (MHT) administered by the Mental Health Commission established under the Act of 2001. Both have the power to discharge, while the MHRB has the additional power to grant conditional discharge. Both the MHRB and MHT consist of a legal chair, a consultant psychiatrist independent of the hospital and a lay member. Patients have a right to legal representation while the treating consultant psychiatrist is called on to explain the reasons for continued detention. The two have a number of legal and procedural differences [11].

At the time of this study there were 83 beds in the Central Mental Hospital, the only forensic hospital for Ireland providing conditions of high, medium and low therapeutic security for patients according to individual need [14,15].

### Statistics

All data were entered first in Excel then in SPSS16 [16] for analysis. Spearman rank correlations were used. Exact binomial probabilities were calculated for differences from an assumed random frequency of 0.5. For the receiver operating characteristic, the area under the curve is calculated with 95% confidence intervals, and significance level is calculated for deviation from a 'random' area under the curve of 0.5.

### Sample

This study was approved by the local research ethics committee. All participants were given an explanation of the project and assured of confidentiality. They were then asked to give signed voluntary consent. Those who consented were asked to complete the WAI and ITP first concerning their assessment of their therapeutic relationship with their consultant psychiatrist, then with their primary nurse.

All 83 were rated in April 2008 (T1) by their consultant psychiatrist and primary nurse for the WAI, ITP, GAF and PANSS. Also in April 2008 a total of 81 patients (of 83, 98%) completed the assessments of WAI and ITP concerning their consultant psychiatrist and their primary nurse (six females of 8, 75%). The PANSS and GAF were also rated by assistant psychologists..

Two weeks prior to their next MHT or MHRB hearing (T2) and two weeks after the hearing (T3) the patients were asked to complete the WAI and ITP again, concerning both their consultant psychiatrist and their primary nurse, as were the treating clinicians. Patients and treating consultant psychiatrists were also asked to complete the MacArthur assessments of perceived coercion, perceived procedural justice and general impact of the hearing. Of the 83 patients assessed at baseline (T1), 75 completed the pre-hearing (T2) assessments. None were discharged by the hearing and the same 75 also completed the post-hearing (T3) assessments. The mean age for this 75 was 41.7 years (SD 12.1), mean time since admission was 6.8 years, (SD 9.4). Diagnoses were schizophrenia 54 (72%), bi-polar affective disorder 4 (5%), psychotic depression 4 (5%), schizoaffective disorder 5 (7%), paranoid psychosis 4 (5%), other 4 (5%). The group who dropped out did not differ significantly from those completing the study in any of the baseline measures or demographic characteristics. Results are presented throughout for the 75 who completed the assessments.

There were 17 patients detained under civil mental health legislation and reviewed by the MHT while 58 were detained under the Criminal Law (Insanity) Act 2001 and accordingly reviewed by the MHRB. There was no difference between the MHT and MHRB groups for age, length of stay, or diagnosis.

A mean of 162.1 days (SD 52.4) elapsed between the baseline (T1) and pre-hearing (T2) assessments and a mean of 32.4 days (SD 31.1) between the pre-hearing (T2) and post hearing (T3) assessments. There were no significant differences in time intervals between those reviewed by MHTs and MHRBs.

### Instruments used

#### Working Alliance Inventory and Interpersonal Trust in Physician

We have previously described the validation of our adaptation of the two scales for measuring therapeutic rapport in forensic mental health settings. The Working Alliance Inventory [8] (additional file 1), is a 12 item self-report questionnaire, designed for completion by patients concerning an individual clinician. Each item is rated on a seven point scale from 'never' to 'always', so that '4' ('sometimes') may be taken as a neutral or non-committal rating. We have drafted modifications so that the tool can also be completed by the treating clinicians (not just by doctors) concerning the patient, using exactly the same wording and exactly the same number of positively and negatively rated items [10].

The Interpersonal Trust in a Physician scale [9] (additional file 1) is a ten item self report questionnaire designed to be completed by patients, about their physicians. Each item is rated 1 to 5 from 'strongly disagree' to 'strongly agree', with '3' representing 'neutral'. We have drafted modifications so that the tool can also be completed by the patient concerning their treating clinicians and by the treating clinicians regarding the patient using exactly the same wording and the same number of positively and negatively rated items [10]. We also administered a version of the ITP in which the patient is asked to rate their attitude to doctors in general.

#### Global function and mental state

The Global Assessment of Function (GAF) [12] and the Positive and Negative Symptom Scale (PANSS) [13] were separately administered at baseline by assistant psychologists trained in the use of those instruments and blind to the WAI and IPT results. These were obtained in April 2008 for all patients as part of their routine clinical assessments. Based on earlier assessments remission status was also established using the criteria of Andreason et al [17].

#### Possible confounding factors

Mental state may bias the subjective perceptions of patients while mental state and global function may also influence the views of clinicians. At baseline (T1, six months before the next hearing) GAF was lower (worse) for those detained under the Mental Health Act 2001 when compared to those detained under the Criminal Law (Insanity) Act (mean 48.3(SD 19.3), n = 17 vs 63.3(18.0), n = 58, ANOVA F = 8.8, df = 73, p = 0.004), the PANSS total score was higher (worse) for those detained under the Act of 2001 (62.5(20.9) vs 50.2(16.6), F = 6.4, p = 0.014) and the PANSS positive symptom score was significantly higher (worse) in those detained under the Act of 2001 (15.5(6.9) v 11.5(5.0) F = 6.9, p = 0.010). PANSS negative and PANSS general symptom scores tended to be higher also in those detained under the Act of 2001 and who would go on to appear before the MHT.

Remission status is defined as scoring below threshold level on eight key symptoms of mental illness on the PANSS and sustaining this status for at least six months [17]. Remission status did not differ significantly at baseline between those detained under either Act. The patients' rating of WAI concerning their consultant psychiatrist was higher (more positive) at baseline (T1, six months before the hearing) in those who were then in remission (n = 20) compared to those who were not in remission (n = 55) (not in remission 55.6(SD 15.6), in remission 66.6(13.9) ANOVA F = 4.9, p = 0.03). This was true also for the patients' rating of ITP concerning their consultant psychiatrist (not in remission 37.4(7.3), in remission 41.7(5.5), ANOVA F = 5.7, p = 0.019). Primary nurses also tended to be rated higher by those in remission though this did not reach statistical significance, and the patients' rating of ITP for doctors in general did not differ between those in remission and those not in remission.

#### Perceived coercion, perceived procedural justice and impact of hearing

The MacArthur scales [2,3] for perceived coercion, perceived procedural justice and impact of hearings were minimally modified for use in this context (Additional file 2).

The MacArthur Perceived Coercion Scale elicits positive or negative appraisals of five items concerning subjective control or subjective coercion over the outcome of the hearing-freedom, choice, initiative, control and influence. Each item is rated from 1 (no person control) to 7 (personal control), so 4 can be taken as neutral. These items were later recoded as dichotomous (0/1), where a rating of four or more counted as positive. Cronbach's alpha was 0.714 for patients and 0.623 for the consultant psychiatrist (0.655 for the dichotomised scale).

The MacArthur Perceived Procedural Justice Scale elicits six items concerning the role of an actor in the hearing. These are *voice *(whether the subject was able to express themselves to the person in question), *interest *(whether the person in question was interested in the subject), *respect*, *fairness*, *satisfaction with the person in question *and *satisfaction with the procedure overall*. These items are rated on a Likert self report scale from 1 ('not at all') to 7 ('definitely'). Patients were asked to rate these items first regarding the legal chair of the hearing and their legal representative, then separately regarding the role of their treating consultant psychiatrist in the hearing. A further scale rated in the same way elicits the impact of the hearing on the patient.

The treating consultant psychiatrist was asked to complete the same scales, including the Perceived Procedural Justice Scale regarding the legal chair of the hearing and patient's legal representative, and separately regarding the patient's role in the hearing. In order to analyse perceived coercion, perceived procedural justice and the impact of the hearing as outcomes, these items were later recoded as dichotmous (0/1), where a rating of '4' or more counted as positive. Cronbach's alpha was 0.904 for the patients rating the legal actors in the hearing (0.820 for the dichotomised scale) and 0.858 for the consultant psychiatrist (0.523 for the dichotomised scale). When the patient rated their treating consultant psychiatrist in the hearing, Cronbach's alpha was 0.925 (dichotomised alpha = 0.871) and when the consultant psychiatrist rated their patient in the hearing Cronbach's alpha was 0.864 (dichotomised alpha = 0.817).

The MacArthur Impact of Hearing Scale elicits ratings for six items regarding the person's subjective feelings after the hearing on self report Likert scales from 1 to 7, *worse/better, upset/calm, less respected/more respected, confused/informed, less hopeful/more hopeful*, and *'globally overall' *good/bad. These items were also later recoded as dichotomous (0/1), where a rating of '4' or more counted as positive. When patients rated the impact of the hearing, Cronbach's alpha was 0.888 (dichotomised alpha = 0.847) and when consultant psychiatrists rated this, alpha = 0.906 (dichotomised alpha = 0.825).

The scales composed of summated dichotomised items generally correlated better than the scales of 'raw' data. The patients' and psychiatrists' ratings of perceived coercion correlated Spearman r = -0.322, p = 0.005. Patients and psychiatrists' ratings of perceived procedural justice regarding the legal actors in the hearing Spearman r = -0.11 (NS). The patients' rating of perceived procedural justice in the hearing concerning their treating psychiatrist's role correlated with the psychiatrists' rating of the patients' role in the same hearing r = 0.695 p < 0.001 (r = 0.304, p = 0.008 for the full scale items summated) and comparing the patients and psychiatrists' rating of the impact of the hearing, r = 0.189 (NS). When the patient and psychiatrist ratings for each dichotomised item were compared, there was a high degree of agreement. For perceived coercion, patient and psychiatrist agreed for three or more items in only 13 out of 75 cases. However, for perceived procedural justice regarding the role of the legal actors in the hearings, patient and psychiatrist agreed for 4 or more of the 6 items in 47 (63%) of 75 cases (binomial exact probability compared to expected random 50% p = 0.037). For perceived procedural justice concerning the roles of patient and psychiatrist, the patient and consultant ratings agreed in 4 or more items in 56 (75%) of 75 cases, (exact binomial probability compared to random 50% p < 0.001). For the impact of the hearing, patient and psychiatrist agreed on four or more items in 55 (73%) of 75 cases (exact binomial probability compared to random 50% p < 0.001). This suggests that the mutual perceptions of patient and psychiatrist regarding the hearing agreed very well concerning the nature of the hearings in specific domains such as perceived procedural justice and impact, but not concerning perceived coercion.

The dichotomised items were next used to divide the overall scale scores for subjective ratings of aspects of the hearings into 'positive' and negative'. For the five item perceived coercion scale (dichotomised) a score of 3 or more was taken as 'positive,' while for the remaining six item scales, a score of 4 or more was taken as 'positive'. Once again, the patient and psychiatrist did not agree regarding perceived coercion, with 12 (16%) of the pairs both rating 'positive' and 17 (23%) of the pairs both rating negative (X^2 ^= 2.6, df = 1, NS). For perceived procedural justice regarding the legal actors in the hearings, the psychiatrist and patient both rated this positive in 59/75 (79%), both negative in none (X^2 ^= 0.3, NS). For perceived procedural justice regarding the role of patient and psychiatrist, the mutual ratings agreed that this was positive in 63 (84%) and negative in 6 (8%), an agreement of 69/75 (92%) overall (X^2 ^= 31.1, p < 0.001). For the impact of the hearing, the patient and psychiatrist both rated this positive in 62 (83%), both rated it negative in 3 (4%), an agreement overall in 65/75 (87%) (X^2 ^= 10.9, p < 0.001, all df = 1, all n = 75 pairs)

## Results

### Relationship between hearings and perceived coercion

**Figure 2:**
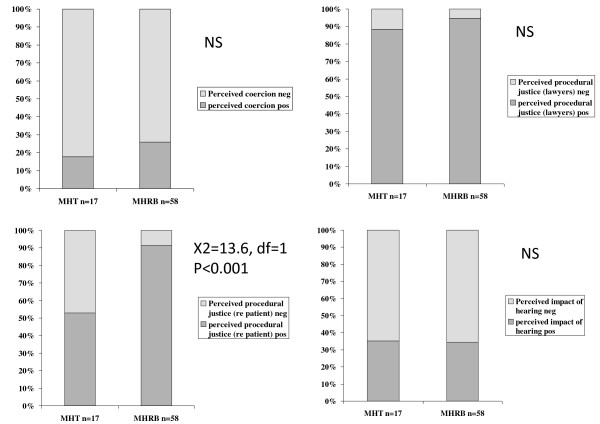
**Consultant psychiatrists' appraisals of two types of hearings**. Consultant psychiatrists' ratings of hearings as coercive, procedural justice regarding the role of legal chair and their own solicitor and procedural justice regarding their psychiatrist, and the impact of the hearing.

There was no significant association between the dichotomised ratings of perceived coercion made by the patients, and whether they appeared before a MHT or MHRB (X^2 ^= 0.9, NS). However the patients' ratings of perceived procedural justice based on the role of the legal chair and the patients' legal representatives, (dichotomised as positive or negative) were associated with the nature of the hearing (X^2 ^= 7.9, df = 1, p = 0.005), with 42 (72%) of the 58 reviewed by the MHRB rating it positively compared to 6 (35%) of 17 attending the MHT. For perceived procedural justice based on the role of the treating consultant psychiatrist in the hearing, again dichotomised as positive or negative, the nature of the hearing was significantly associated with the appraisal (Chi-sq = 10.8, df = 1, p < 0.001) with 45 (78%) of 58 appearing before the MHRB rating the role of the psychiatrist positively compared to 6 (35%) of 17 appearing before the MHT. Patients rated the impact of the hearing as positive or negative in the same way, (Chi-sq = 7.9, df = 1, p = 0.005) with 42 (72%) of 58 appearing before the MHRB again rating the hearing positively compared to 6 (35%) of 17 rating the MHT (Figure 1). Figure 2 shows the same ratings of the two types of hearings made by the consultant psychiatrists.

### WAI and ITP before and after hearings

**Figure 3:**
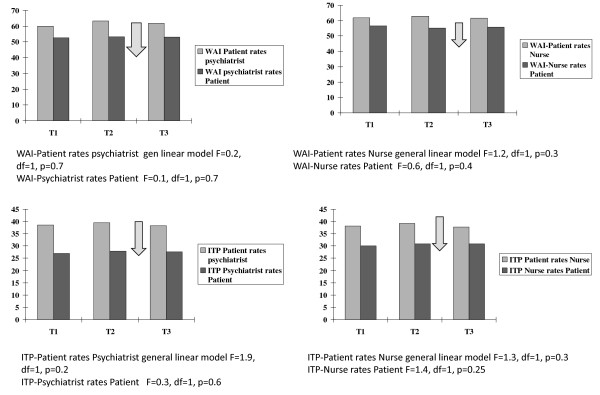
**WAI and ITP before and after hearings**. Note that the arrows indicate the time of the hearings.

**Figure 4:**
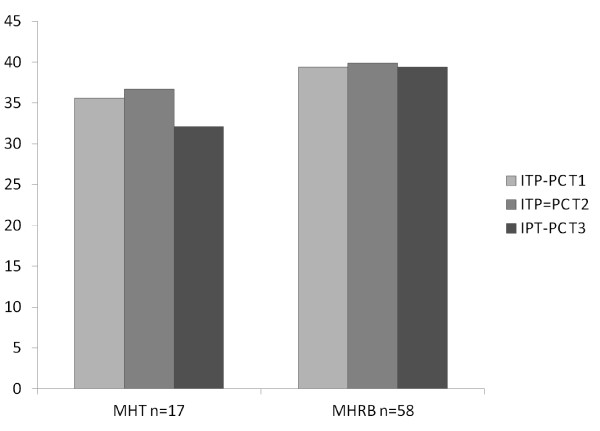
**MHT and MHRB groups compared**. Results are for the ITP when patients rated their consultant psychiatrists.

Table 1 and Figure 3 shows that the WAI and ITP did not change significantly between the baseline six months before a hearing (T1) and the assessment (T2) two weeks prior to the next hearing (paired t-tests for all pairs). Most ratings did not change when comparing before (T2) and after the hearing (T3). The patient ratings of Interpersonal Trust (ITP) for their consultant psychiatrist and for their primary nurse were significantly lower when comparing scores at T2 and T3 i.e. comparing two weeks before and two weeks after the hearings ITP (patient rates consultant), mean difference = 1.4 (SD 6.0), paired t = 2.1, df = 74, p = 0.042; ITP (patient rates nurse) mean difference = 1.5 (SD 5.8), paired t = 2.3, df = 74, p = 0.027. This difference appears to arise mainly from those patients who had been before the MHT as there were no significant differences for those who had attended the MHRB. For the 17 patients before the MHT, ITP (patient rates consultant) mean difference = 4.5 (SD 8.3), paired t = 2.3, df = 16, p = 0.038 Figure 4). However ITP (patient rates nurse) mean difference = 2.4 (SD7.0), paired t = 1.4, NS. All other paired differences for the MHT and MHRB comparing T2 and T3 were not significant.

**Table 1:** Working Alliance Inventory (WAI) and Interpersonal Trust in Physician (ITP) six months before a hearing (T0), two weeks before (T1) and two weeks after (T2) a hearing.

	Mental Health Tribunal n = 17			**Mental Health Review Board**, n = 58			All n = 75		
	**T1**	**T2**	**T3**	**T1**	**T2**	**T3**	**T1**	**T2**	**T3**

Patient rates consultant psychiatrist									

WAI	57.2(14.9)	56.3(18.2)	51.2(21.8)	60.8(15.8)	64.7(17.5)	64.0(15.8)	59.9(15.6)	63.3(17.2)	61.1(17.9)

ITP	35.6(7.1)	36.7(7.7)	32.1(9.0)	39.4(6.9)	39.9(8.0)	39.4(8.7)	38.5(7.1)	39.5(7.6)	37.8(9.2)

Patient rates primary nurse									

WAI	58.6(16.7)	56.7(19.2)	51.6(19.9)	63.1(13.7)	64.3(16.4)	63.7(15.5)	61.9(14.5)	62.9(16.5)	60.9(17.2)

ITP	36.4(6.5)	36.3(6.9)	33.9(7.6)	38.6(5.0)	39.6(8.2)	38.4(8.4)	38.1(5.5)	39.2(7.5)	37.4(8.4)

Patient rates doctors in general									

ITP	40.2(7.6)	37.7(6.4)	35.8(8.7)	40.1(6.8)	41.3(8.9)	41.4(8.6)	40.1(6.9)	40.9(7.9)	40.2(8.9)

Consultant psychiatrist rates patient									

WAI	43.7(15.7)	43.9(18.3)	41.5(18.2)	55.1(15.0)	55.8(14.0)	56.5(15.1)	52.6(15.8)	53.2(15.8)	53.1(16.9)

ITP	22.0(8.1)	21.2(6.3)	19.2(7.1)	28.5(8.6)	29.8(8.1)	30.0(9.3)	27.0(8.9)	27.9(8.5)	27.6(9.9)

Primary nurse rates patient									

WAI	51.3(10.3)	48.7(14.2)	48.4(14.6)	58.2(13.4)	57.1(13.9)	57.9(12.3)	56.6(13.0)	55.2(14.3)	55.8(13.3)

ITP	24.8(4.1)	26.4(5.5)	26.9(7.8)	31.6(7.2)	32.2(7.4)	31.9(7.9)	30.0(7.2)	30.9(7.4)	30.8(8.1)

Means and standard deviations.

### MHTs and MHRBs compared

Table 2 shows that at each time point, the ratings of WAI and ITP were lower for those reviewed by the MHT, i.e. those transferred from other hospitals under the civil Mental Health Act 2001, than for those reviewed by the MHRB i.e. those detained under the Criminal Law (Insanity) Act 2006. This was true even at T1, six months before the next hearing. To distinguish between the effects of time (before and after the hearing) and the nature of the hearing (MHT or MHRB), a general linear model for univariate analysis of variance was used, with each scale as the dependant variable and two fixed factors, time (T1, T2 and T3) and hearing (MHT or MHRB). For each scale (WAI and ITP, concerning consultant psychiatrists and primary nurses rated by patients, consultant psychiatrists and primary nurses, and the ITP concerning the patient's appraisal of doctors in general), time had no significant effect but the hearing had a significant effect (for the effect of the hearing, F ranged from 5.6, p = 0.019 to 27.4, p < 0.001). This confirms that those undergoing MHTs had lower scores for WAI and ITP before as well as after their hearings, with no significant change after the hearing itself on this analysis. Figure 4 illustrates this for the ITP when patients rated their consultant psychiatrists.

**Table 2:** WAI and ITP according to the legal category of detention.

	T1		T2		T3	
	**Mental Health Act 2001**	**Criminal Law (Insanity) Act 2006**	**Mental Health Act 2001**	**Criminal Law (Insanity) Act 2006**	**Mental Health Act 2001**	**Criminal Law (Insanity) Act 2006**

Patient rates consultant psychiatrist						

WAI	57.2(14.9)	60.8(15.8)	56.3(18.2)	64.7(17.5)	51.2(21.8)	64.0(15.8)

ITP	35.6(7.1)	39.4(6.9)	36.7(7.7)	39.9(8.0)	32.1(9.0)	39.4(8.7)

Patient rates primary nurse						

WAI	58.1(16.7)	63.1(13.7)	56.7(19.2)	64.3(16.4)	51.6(19.9)	63.7(15.5)

ITP	36.4(6.5)	38.6(5.0)	36.3(6.9)	39.6(8.2)	33.9(7.6)	38.4(8.4)

Patient rates doctors in general						

ITP	40.2(7.6)	40.1(6.8)	37.7(6.4)	41.3(8.9)	35.8(8.7)	41.4(8.6)

Consultant psychiatrist rates patient						

WAI	43.7(15.7)	55.1(15.0)	43.9(18.3)	55.9(14.0)	41.5(18.2)	56.5(15.1)

ITP	22.0(8.1)	28.5(8.7)	21.2(6.3)	29.8(8.1)	19.2(7.1)	30.0(9.3

Primary nurse rates patient						

WAI	51.3(10.3)	58.2(13.4)	48.7(12.2)	57.1(13.9)	48.4(14.6)	57.9(12.3)

ITP	24.8(4.1)	31.6(7.2)	26.4(5.5)	32.2(7.4)	29.9(7.8)	31.9(7.9)

Note that those detained under the Mental Health Act 2001 must be reviewed by the Mental Health Tribunal (n = 17), those detained under the Criminal Law (Insanity) Act 2006 must be reviewed by the Mental Health Review Board (n = 58). T1 = six months before the next hearing, T2 = two weeks before and T3 = two weeks after the hearing. Means and standard deviations.

### Perceived coercion, perceived procedural justice and impact of hearing on WAI and ITP

To test the hypothesis that the conduct of the hearing itself (or the perception of it) might have a positive or negative effect on working alliance and interpersonal trust, the MacArthur scales completed by the patients immediately after the hearings were used as described above to categorise the patients' perceived coerciveness of the hearing as positive or negative. Similarly, they were used for the patients' perceived procedural justice in relation to the legal chair and legal representative, the consultant psychiatrist's role in the hearing, and the impact of the hearing.

**Figure 5:**
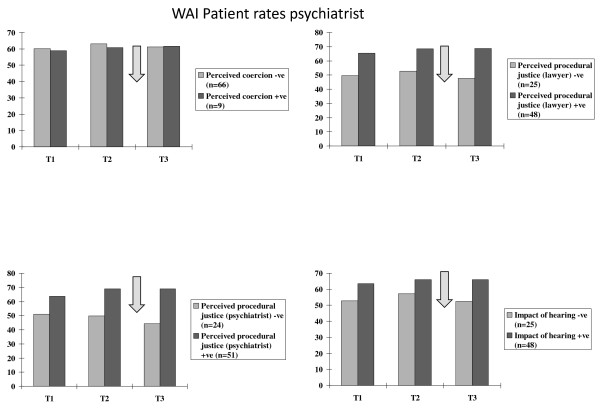
**Patients' perceptions of the hearing and WAI patient ratings of psychiatrist before and after the hearing**. Note that the arrows indicate the time of the hearings.

**Figure 6:**
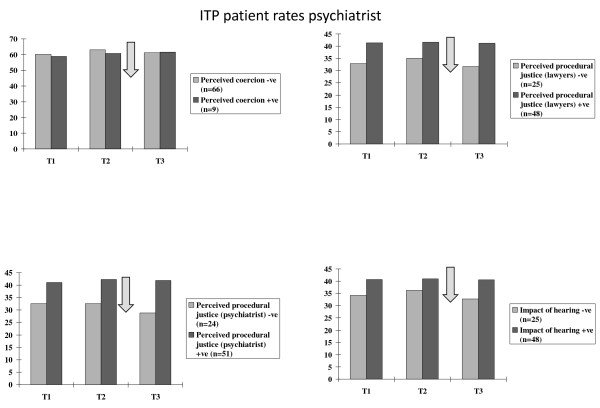
**Patients' perceptions of the hearing and patient ratings of ITP re their psychiatrist before and after the hearing**. Note that the arrows indicate the time of the hearings.

Table 3 shows that perceived coercion did not substantially influence the patients' rating of WAI or ITP with their treating consultant, their primary nurse or doctors in general, with stability over the three time points from six months before to two weeks after the hearing. Tables 4, 5 and 6 however show that in general, those who rated the hearing as a negative experience from the point of view of perceived procedural justice concerning the role of the legal chair of the hearing and the patient's legal representative (table 4), or perceived procedural justice concerning the role of the treating consultant psychiatrist (table 5) or an overall appraisal of the impact of the hearing (table 6) also rated the WAI and ITP negatively concerning their treating consultant psychiatrist. However this could not be causally attributed to the hearing-those who went on to make negative appraisals of the hearing had already made negative appraisals of their working alliance and interpersonal trust with their treating consultant, six months before and two weeks before the hearing (Figures 5 &6). Further, those making negative appraisals of the hearing also made negative ratings of their working alliance and interpersonal trust with their primary nurse, who was not directly involved in the hearing, and with doctors in general, in each case before as well as after the hearing.

**Table 3:** Working Alliance Inventory (WAI) and Interpersonal Trust in Physician (ITP) six months before a hearing (T0), two weeks before (T1) and two weeks after (T2) a hearing according to dichotomised patients' ratings of perceived coercion regarding the hearing, n = 75.

	T1: six months before hearing			T2: two weeks before hearing			T3: two weeks after hearing		
	**Perceived Coercion**								

	**Negative** **N = 49**	**Positive** **N = 26**	**ANOVA** **F/p**	**Negative** **N = 49**	**Positive** **N = 26**	**ANOVA** **F/p**	**Negative** **N = 49**	**Positive** **N = 26**	**ANOVA** **F/p**

Patient rates consultant psychiatrist									

WAI	59.3(15.0)	61.1(16.9)	0.2/NS	61.4(18.9)	65.6(15.7)	0.9/NS	58.3(18.6)	66.4(15.7)	3.6/NS

ITP	37,7(6.9)	39.9(7.3)	1.6/NS	38.5(8.2)	40.5(7.6)	1.1/NS	36.6(9.4)	40.0(8.7)	2.4/NS

Patient rates primary nurse									

WAI	61.6(14.1)	62.7(15.5)	0.1/NS	61.4(18.5)	64.7(14.7)	0.6/NS	58.6(18.2)	65.4(14.6)	2.7/NS

ITP	37.7(5.7)	39.0(4.9)	1.0/NS	38.1(8.2)	40.4(7.5)	1.4/NS	36.2(8.8)	39.6(7.3)	2.9/NS

Patient rates doctors in general									

ITP	39.9(6.6)	40.4(7.7)	0.1/NS	39.2(8.9)	42.9(7.4)	3.1/NS	38.6(9.1)	43.1(7.9)	4.7/0.034

Means and standard deviations.

**Table 4:** Working Alliance Inventory (WAI) and Interpersonal Trust in Physician (ITP) six months before a hearing (T0), two weeks before (T1) and two weeks after (T2) a hearing according to dichotomised patients' ratings of perceived procedural justice concerning the roles of the legal chair and the patient's legal representative in the hearing n = 75.

	T1: six months before hearing			T2: two weeks before hearing			T3: two weeks after hearing		
	**Patient's Rating of Perceived Procedural Justice (Legal)**								

	**Negative** **N = 15**	**Positive** **N = 60**	**ANOVA** **F/p**	**Negative** **N = 15**	**Positive** **N = 60**	**ANOVA** **F/p**	**Negative** **N = 15**	**Positive** **N = 60**	**ANOVA** **F/p**

Patient rates consultant psychiatrist									

WAI	45.9(13.5)	63.3(14.3)	17.0/0.001	48.3(19.6)	66.5(15.6)	14.7/0.001	41.9(15.9)	65.9(15.1)	29.9/0.001

ITP	31.4(5.5)	40.2(6.4)	22.4/0.001	32.8(7.8)	40.8(7.3)	14.0/0.001	27.2(8.4)	40.4(7.4)	36.4/0.001

Patient rates primary nurse									

WAI	52.3(15.4)	64.3(13.5)	8.5/0.005	45.9(20.7)	66.7(13.5)	22.8/0.001	42.1(19.6)	65.7(13.0)	31.5/0.001

ITP	33.5(6.5)	39.2(4.6)	14.7/0.001	32.1(9.2)	45.6(6.7)	16.4/0.001	27.2(7.7)	39.9(6.5)	42.6/0.001

Patient rates doctors in general									

ITP	37.8(8.3)	40.7(6.5)	1.9/NS	34.0(8.5)	42.1(7.8)	12.6/0.001	30.5(7.2)	42.6(7.6)	31.4/0.001

Means and standard deviations.

**Table 5:** Working Alliance Inventory (WAI) and Interpersonal Trust in Physician (ITP) six months before a hearing (T0), two weeks before (T1) and two weeks after (T2) a hearing according to dichotomised patients' ratings of perceived procedural justice concerning the role of the treating consultant psychiatrist in the hearing.

	T1: six months before hearing			T2: two weeks before hearing			T3: two weeks after hearing		
	**Patient's Rating of Perceived Procedural Justice (Medical)**								

	**negative**	**positive**	**ANOVA** **F/p**	**negative**	**positive**	**ANOVA** **F/p**	**negative**	**Positive**	**ANOVA** **F/p**

Patient rates consultant psychiatrist									

WAI	48.8(12.8)	61.9(15.3)	7.1/0.009	46.6(14.8)	65.6(16.9)	12.1/0.001	38.0(13.8)	65.1(15.5)	29.5/0.001

ITP	32.6(4.8)	39.5(6.9)	9.9/0.002	32.6(6.6)	40.3(7.7)	9.9/0.002	26.2(8.5)	39.8(7.8)	27.7/0.001

Patient rates primary nurse									

WAI	47.8(16.5)	64.5(12.7)	14.6/0.001	46.3(16.1)	65.3(15.9)	13.5/0/001	39.1(17.4)	64.7(14.3)	28.3/0.001

ITP	33.0(6.9)	39.0(4.7)	13.3/0.001	33.1(6.9)	39.9(7.8)	7.3/0.008	26.6(4.4)	39.2(7.5)	28.7/0.001

Patient rates doctors in general									

ITP	39.1(8.5)	40.3(6.7)	0.3/NS	37.4(4.8)	41.0(8.9)	1.8/NS	31.6(6.1)	41.6(8.5)	14.2/0.001

n = 75. Means and standard deviations.

**Table 6:** Working Alliance Inventory (WAI) and Interpersonal Trust in Physician (ITP) six months before a hearing (T0), two weeks before (T1) and two weeks after (T2) a hearing according to dichotomised patients' ratings of impact of the hearing.

	T1: six months before hearing			T2: two weeks before hearing			T3: two weeks after hearing		
	**Patient's Rating of Perceived Impact of Hearing**								

	**Negative**	**positive**	**ANOVA** **F/p**	**negative**	**positive**	**ANOVA** **F/p**	**negative**	**Positive**	**ANOVA** **F/p**

Patient rates consultant psychiatrist									

WAI	51.9(14.9)	61.4(15.4)	3.5/NS	49.5(16.5)	65.4(17.1)	8.7/0.004	45.4(17.7)	64.1(16.5)	12.6/0.001

ITP	33.6(7.8)	39.4(6.6)	11.3/0.011	33.1(8.2)	40.4(7.5)	9.2/0.003	28.9(9.5)	39.4(8.2)	15.8/0.001

Patient rates primary nurse									

WAI	49.3(16.3)	64.2(13.1)	11.3/0.001	45.3(18.6)	65.8(14.9)	17.6/0.001	43.6(21.5)	64.3(14.3)	17.8/).001

ITP	32.2(5.4)	39.2(4.8)	19.2/0.001	32.3(9.6)	40.1(7.1)	10.8/0.002	28.4(9.5)	39.1(7.1)	20.3/0.001

Patient rates doctors in general									

ITP	37.4(7.6)	40.6(6.7)	2.1/NS	34.9(9.4)	41.6(7.9)	6.6/0.012	29.8(8.5)	42.1(7.5)	26.2/0.001

n = 75. Means and standard deviations.

Using a general linear model for univariate analysis of variance, taking the patients' rating of either WAI or ITP concerning their treating consultant psychiatrist as the dependent factor and two fixed factors, time (T1, T2 or T3) and the dichotomised rating of perceived coercion (table 3), neither time, perceived coercion nor the interaction between the two fixed factors reached significance.

For perceived procedural justice regarding the role of the legal chair and legal representative (table 4), neither time nor appraisal alone were significant but the interaction between time and negative perception was significant for the WAI (F = 6.1, p = 0.003) and ITP (F = 6.6, p = 0.002) concerning the consultant psychiatrist (Figures 5 &6).

For perceived procedural justice regarding the role of the treating psychiatrist (table 5), only the interaction for the ITP between time and negative appraisal reached significance (F = 4.7, p = 0.010) (Figure 6).

Finally, for the patients' appraisal of the impact of the hearing (table 6) only the interaction for the ITP between time and the patients' appraisal of the hearing reached significance (F = 3.3, p = 0.041) (Figure 6).

### Counfounding factors: baseline (T1) effect of WAI and ITP, GAF and PANSS on perceived coercion, perceived procedural justice and impact of hearing

**Figure 7:**
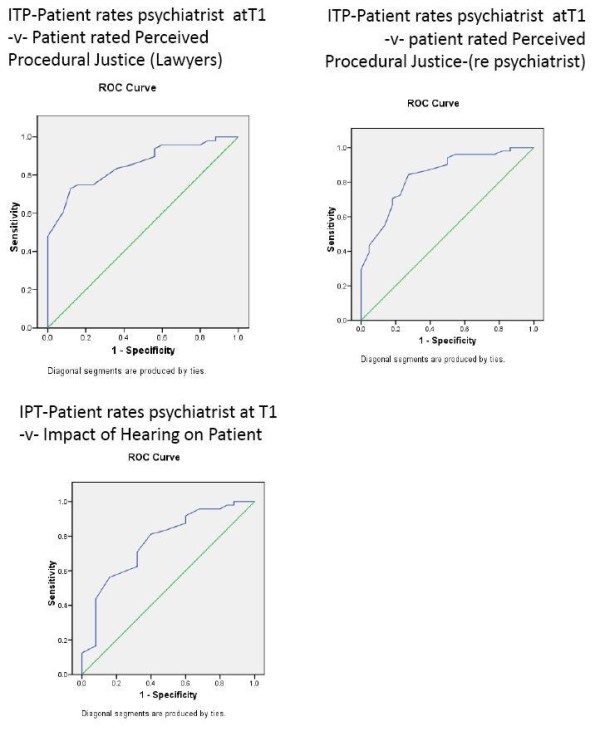
**ROC curves for patient-rated ITP re their psychiatrist at T1 and the patient's positive or negative perceptions of the hearing**.

**Figure 8:**
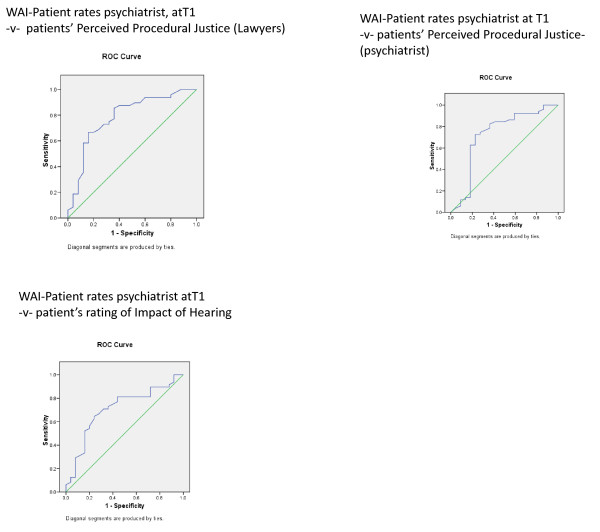
**ROC curves for patient-rated WAI re their psychiatrist at T1 and the patient's positive or negative perceptions of the hearing**.

Table 7 shows that the patients' rating of working alliance and interpersonal trust for their treating consultant psychiatrist six months prior to the hearing predicted their subsequent rating of the hearing for perceived procedural justice regarding the role of the legal chair and legal representative, perceived procedural justice regarding the role of the treating consultant psychiatrist and the impact of the hearing, though not for the patients' rating of perceived coercion (Figures 7 and 8). Of note, the patients' baseline rating of WAI and ITP regarding their primary nurse also predicted their eventual ratings of perceived procedural justice and impact of the hearing, though the patients' baseline rating of ITP concerning doctors in general did not predict the patients' eventual appraisal of the hearing.

**Table 7:** Receiver Operating Characteristic (ROC) area under the curve (AUC) for dichotomised perceived coercion, perceived procedural justice and impact of the hearing as predicted by patients' ratings of Working Alliance Inventory (WAI) and Interpersonal Trust in Physician (ITP) six months before the hearing (T1), null hypothesis: true area under the curve = 0.5.

	AUC	95% CI		P
		**Lower bound**	**Upper bound**	

**Dependent variable: Patient's Perceived Coercion (dichotomised)**				

Patient rates consultant psychiatrist				

WAI	0.554	0.412	0.696	NS

ITP	0.625	0.485	0.764	NS

Patient rates primary nurse				

WAI	0.529	0.382	0.676	NS

ITP	0.625	0.485	0.764	NS

Patient rates doctors in general				

ITP	0.538	0.392	0.885	NS

**Dependent variable: Patient's perceive procedural justice re legal actors in hearing (dichotomised)**				

Patient rates consultant psychiatrist				

WAI	0.828	0.696	0.960	< 0.001

ITP	0.857	0.762	0.952	< 0.001

Patient rates primary nurse				

WAI	0.732	0.578	0.886	0.007

ITP	0.795	0.649	0.941	0.001

Patient rates doctors in general				

ITP	0.659	0.487	0.830	NS

**Dependent variable: Patients' perceived procedural justice re consultant psychiatrist's role in hearing (dichotomised)**				

Patient rates consultant psychiatrist				

WAI	0.772	0.616	0.928	0.004

ITP	0.815	0.705	0.925	0.001

Patient rates primary nurse				

WAI	0.798	0.628	0.968	0.002

ITP	0.795	0.649	0.941	0.001

Patient rates doctors in general				

ITP	0.659	0.487	0.830	NS

**Dependent variable: Patients' perceived impact of the hearing (dichotomised)**				

Patient rates consultant psychiatrist				

WAI	0.697	0.508	0.886	0.038

ITP	0.735	0.548	0.923	0.013

Patient rates primary nurse				

WAI	0.779	0.605	0.953	0.003

ITP	0.830	0.703	0.957	0.001

Patient rates doctors in general				

ITP	0.656	0.475	0.837	NS

n = 75. Means and standard deviations.

**Figure 9:**
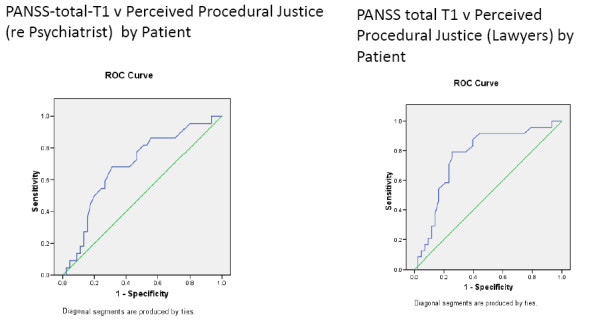
**ROC curves for PANSS total score at T1 and the patient's positive or negative perceptions of the hearing**.

Similarly, the GAF and PANSS scores, rated by psychology assistants blind to other measures, six months prior to the hearing, tended to predict the patients' eventual appraisals of the hearings (table 8 and Figure 9). In keeping with this, remission status [17] at baseline (T1) was not related to patient's appraisal of the hearing in terms of perceived coercion (dichotomised as positive or negative, X2 = 0.3, df = 1, NS), but remission status at baseline (T1) was associated with perceived procedural justice concerning the role of the legal actors in the hearing (19 (95%) of 20 in remission rated the legal actors positively compared to 41 (75%) of 55 not in remission, X2 = 3.8, p = 0.05), perceived procedural justice regarding the consultant psychiatrists' role in the hearing (all 20 in remission rated the role of the psychiatrist positively compared to 42 (80%) of 55 not in remission, X2 = 4.7, p = 0.03) while remission status was not significantly related to the impact of the hearing.

**Table 8:** Receiver Operating Characteristic (ROC) area under the curve (AUC) for dichotomised perceived coercion, perceived procedural justice and impact of the hearing as predicted by independently rated Global Assessment of Function and PANSS scales six months before the hearing (T1), null hypothesis: true area under the curve = 0.5.

	AUC	95% CI		P
		**Lower bound**	**Upper bound**	

Dependent variable: Patient's Perceived Coercion (dichotomised)				

GAF	0.467	0.330	0.604	NS

PANSS total score	0.534	0.392	0.676	NS

PANSS positive	0.460	0.324	0.595	NS

PANSS negative	0.573	0.434	0.711	NS

PANSS general	0.492	0.354	0.629	NS

Dependent variable: Patient's perceived procedural justice re legal actors in hearing (dichotomised)				

GAF	0.810	0.714	0.907	< 0.001

PANSS total score	0.807	0.709	0.904	< 0.001

PANSS positive	0.711	0.567	0.856	0.014

PANSS negative	0.779	0.675	0.882	0.001

PANSS general	0.509	0.318	0.699	NS

Dependent Variable: Patients' perceived procedural justice re consultant psychiatrist's role in hearing (dichotomised)				

GAF	0.802	0.670	0.935	0.001

PANSS total score	0.837	0.738	0.936	< 0.001

PANSS positive	0.828	0.668	0.987	< 0.001

PANSS negative	0.738	0.621	0.856	0.012

PANSS general	0.530	0.354	0.704	NS

Dependent variable: Patients' perceived impact of the hearing (dichotomised)				

GAF	0.673	0.511	0.835	NS

PANSS total score	0.721	0.588	0.854	0.016

PANSS positive	0.677	0.503	0.852	NS

PANSS negative	0.608	0.457	0.759	NS

PANSS general	0.588	0.410	0.766	NS

n = 75. Means and standard deviations.

## Discussion

There has been considerable interest amongst lawyers and clinicians in the concept of therapeutic jurisprudence for the mentally disordered. This hypothesises that a less coercive, more transparent and fair legal process may have beneficial effects on longer term treatment adherence and outcomes generally [1]. There has been supporting evidence for this in respect of committal hearings [2,3].

### Studying perceptions

The content of the scale used to measure perceived coercion in the hearing was designed for a committal hearing, rather than a review hearing, and did not work well in this study of review hearings. We have reported the results using this rating, alongside the ratings of perceived procedural justice concerning legal and medical roles and the impact of the hearing, as a sort of control. In the same way, the patient's ratings of WAI and ITP concerning the primary nurse represents a 'control' for their ratings of WAI and ITP concerning their treating consultant psychiatrist, as the primary nurse was not directly involved in the hearing. The rating of ITP concerning doctors in general is likewise a 'control'.

A point of methodological interest is the distinction between correlation using the Spearman rank correlation coefficient or the Chi-squared test, and exact binomial probabilities for agreement. We found that correlation between psychiatrist and patient concerning the hearings appeared weak, even though they agreed in a high proportion of cases. Therefore, the use of binomial exact probabilities for rates of agreement is a better way of assessing concordance between patient and clinician.

### Agreement between patients and clinicians

We have previously reported that there is a considerable degree of 'halo effect' in the patients' ratings of treating psychiatrist and primary nurse, just as there is a 'consensus' correlation between the ratings of psychiatrist and nurse, and a confounding effect due to mental state [10]. It is notable however that the treating psychiatrists' rating of hearings agreed well with the patients' ratings of the hearings whether positive or negative. This suggests that MHTs really were more negative in the ways they are conducted by lawyers and more negative than MHRBs in the roles given to the treating consultant psychiatrist and the patient, and in their overall impact on all concerned. If these differences are objective, or at least shared perceptions, their origins are complex, since the patients' lawyers are apparently following the instructions given by their clients, whether positive or negative. There should be an onus on the legal chair in managing the hearing, to prevent the use of styles of rhetoric or expressed emotion that may tend to further polarise or damage therapeutic relationships for the future.

A cross-sectional reading of the WAI and ITP ratings after the hearing would show much lower ratings for those who had been reviewed by the MHT. The prospective data however show that the differences between patients reviewed by MHTs and MHRBs were present as much as six months prior to the hearing. The prospective data show no evidence for improvement in WAI or ITP after positively rated hearings and only equivocal evidence for worsening after negatively rated hearings when ratings of WAI and ITP six months and two weeks before the hearing are taken into account.

### Mental State as a confounding factor or source of bias

We have previously shown that the patients' ratings of WAI and ITP, like the clinicians' ratings, are correlated with global function (GAF) and symptom severity (PANSS) [10]. This held true for remission status, with those in remission at baseline rating WAI and ITP higher for their treating consultant psychiatrist, but not for the primary nurse or doctors in general. This confounding effect of mental state and global function offers a rational explanation for the differences in rating of WAI and ITP, since those improving with treatment are likely to have more confidence in their treating psychiatrist. The effect extended to the later rating of the hearings as positive or negative, with those in remission more likely to rate the hearings as positive, even though they were not more likely to be discharged. In this time period, no patients were discharged by either MHT or MHRB.

A complex causal model could be constructed from the various inter-correlations and interactions in this data set. Statistical analysis of so many factors would however require a sample of a size that probably cannot be achieved except in a multi-centre study. At the least, this study might represent a pilot for such a project. The clearest signal regarding causation in this data set is contained in tables 7 and 8. Baseline WAI and ITP predicted the eventual appraisal of the hearing by the patient, as did baseline measures of global function (GAF) and symptom severity (PANSS). The appraisal of the hearing was not merely subjective, since the psychiatrist and the patient agreed much more often than by chance. Where patients were not in remission, the role of the legal actors and the role of the clinician-patient pairs were perceived negatively. The patient's lawyer pursuing the patient's instructions must therefore have mediated the negative experience for patient and psychiatrist.

## Conclusions

Based on the findings in this study, we can find little support for improving therapeutic relationships through the conduct of mental health review hearings, but some evidence that negatively perceived hearings can have adverse effects on therapeutic relationships. The best strategy for improved therapeutic relationships continues to be efforts to reduce symptom severity, improve global function and work towards both remission and recovery.

## Competing interests

The authors declare that they have no competing interests.

## Authors' contributions

VD and AL collected the data. DM advised on the literature and design of the study. HGK devised the modified instruments and the study protocol. All authors contributed to the authorship of the paper and approved the final manuscript.

## Supplementary Material

Additional file 1 **Working Alliance Inventory (WAI) **[8]**and Interpersonal Trust in Physician (ITP) **[9]**adapted for patients and clinicians **[10]. Click here for file

Additional file 2 **MacArthur Scales for Perceived Coercion, Procedural Justice and Impact of Hearing adapted for mental health hearings **[2,3]. Click here for file
