# Supplementary material for: Working alliance, interpersonal trust and perceived coercion in mental health review hearings
Source: Int J Ment Health Syst. 2011 Nov 10;5:29. doi: 10.1186/1752-4458-5-29 (PMC3227564; doi:10.1186/1752-4458-5-29)
Supplement: Additional file 1 — Working Alliance Inventory (WAI) [8]and Interpersonal Trust in Physician (ITP) [9]adapted for patients and clinicians [10]. [file 1752-4458-5-29-S1.DOC]

# Additional files

### Additional file 1 – Working Alliance Inventory (WAI) [8] and Interpersonal Trust in Physician (ITP) [9] adapted for patients and clinicians [10]

**Modified WAI [8] (for Clinicians)**– Short form

Instructions Below there are statements which describe some of the different ways a therapist might think or feel about his or her patient. After each statement there is a seven point scale:

1 2 3 4 5 6 7

NEVER RARELY OCCASIONALLY SOMETIMES OFTEN VERY OFTEN ALWAYS

If the statement describes the way you always feel (or think) circle the number 7; if it never applies to you circle the number 1. Use numbers in between to describe the variations between these extremes. Work quickly: your first impressions are the ones we would like to see. PLEASE DO NOT FORGET TO RESPOND TO EVERY ITEM. Thank you for your cooperation.

1. The patient and I agree about things we will need to do in therapy to improve his/her situation.

1 2 3 4 5 6 7

2. What we are doing in therapy gives him/her new ways of looking at his/her problem.

1 2 3 4 5 6 7

3. I believe the patient likes me.

1 2 3 4 5 6 7

4. The patient does not understand what we are trying to accomplish in therapy.

1 2 3 4 5 6 7

5. The patient is confident in my ability to help him/her.

1 2 3 4 5 6 7

6. The patient and I are working towards mutually agreed upon goals.

1 2 3 4 5 6 7

7. I feel that the patient appreciates me.

1 2 3 4 5 6 7

8. We agree on what is important for the patient to work on.

1 2 3 4 5 6 7

9. The patient and I trust one another.

1 2 3 4 5 6 7

10. The patient and I have different ideas on what his/her problems are.

1 2 3 4 5 6 7

11. We have established a good understanding of the kind of changes that would be good for him/her.

1 2 3 4 5 6 7

12. I believe the patient and I have a good working relationship.

1 2 3 4 5 6 7

Date__________ Patient’s Code______________________________

**Modified WAI** **[8]** – Short form (**Patient rates treating psychiatrist /Nurse version**)

Psychiatrist/ Primary Nurse Code

Instructions Below there are statements which describe some of the different ways a therapist might think or feel about his or her patient. After each statement there is a seven point scale:

1 2 3 4 5 6 7

NEVER RARELY OCCASIONALLY SOMETIMES OFTEN VERY OFTEN ALWAYS

If the statement describes the way you always feel (or think) circle the number 7; if it never applies to you circle the number 1. Use numbers in between to describe the variations between these extremes. Work quickly: your first impressions are the ones we would like to see. PLEASE DO NOT FORGET TO RESPOND TO EVERY ITEM. Thank you for your cooperation.

--------------------------------------------------------------------------------------------

1. The Consultant/Nurse and I agree about things we will need to do in therapy to improve my situation.

1 2 3 4 5 6 7

2. What I am doing in therapy gives me new ways of looking at my problem.

1 2 3 4 5 6 7

3. I believe the Consultant/Nurse likes me.

1 2 3 4 5 6 7

4. The Consultant/Nurse does not understand what I am trying to accomplish in therapy.

1 2 3 4 5 6 7

5. I am confident in the Consultant/Nurse’s ability to help me.

1 2 3 4 5 6 7

6. The Consultant/Nurse and I are working towards mutually agreed upon goals.

1 2 3 4 5 6 7

7. I feel that the Consultant/Nurse appreciates me.

1 2 3 4 5 6 7

8. We agree on what is important for me to work on.

1 2 3 4 5 6 7

9. The Consultant/Nurse and I trust one another.

1 2 3 4 5 6 7

10. The Consultant/Nurse and I have different ideas on what my problems are.

1 2 3 4 5 6 7

11. We have established a good understanding of the kind of changes that would be good for me.

1 2 3 4 5 6 7

12. I believe the way we are working with my problem is correct.

1 2 3 4 5 6 7

**Interpersonal Trust in a Physician (for patient) [9]**

10 items, scored 5-1, for Strongly Agree, Agree, Neutral, Disagree, Strongly Disagree. Negatively worded items (2,3,8) are reverse scored.

1. [Your doctor] will do whatever it takes to get you all the care you need.

1 2 3 4 5

2. Sometimes [your doctor] cares more about what is convenient for [him/her] than about your medical needs.

1 2 3 4 5

3. [Your doctor] 's medical skills are not as good as they should be.

1 2 3 4 5

4. [Your doctor] is extremely thorough and careful.

1 2 3 4 5

5. You completely trust [your doctor's] decisions about which medical treatments are best for you.

1 2 3 4 5

6. [Your doctor] is totally honest in telling you about all of the different treatment options available for your condition.

1 2 3 4 5

7. [Your doctor] only thinks about what is best for you.

1 2 3 4 5

8. Sometimes [your doctor] does not pay full attention to what you are trying to tell [him/her]

1 2 3 4 5

9. You have no worries about putting your life in [your doctor] 's hands.

1 2 3 4 5

10. All in all, you have complete trust in [your doctor].

1 2 3 4 5

**Interpersonal Trust in a Patient [9] (for clinicians)**

10 items, scored 5-1, for Strongly Agree, Agree, Neutral, Disagree, Strongly Disagree. Negatively worded items (2,3,8) are reverse scored.

1. [Your patient] will do whatever it takes to benefit from all the care he/she needs.

1 2 3 4 5

2. Sometimes [your patient] cares more about what is convenient for [him/her] than about his/her medical needs.

1 2 3 4 5

3. [Your patient]'s general and mental health knowledge and motivation is not as good as it should be.

1 2 3 4 5

4. [Your patient] is extremely thorough and careful regarding their treatments e.g. fully complies with medication, attends all group and individual activities on offer.

1 2 3 4 5

5. You completely trust [your patient’s] decisions about which medical treatments are best for him or her.

1 2 3 4 5

6. [Your patient] is totally honest in telling you about their preferences and intentions regarding all of the different treatment options available for their condition.

1 2 3 4 5

7. [Your patient] only thinks about what is best for his or her health e.g. in relation to substance misuse or side effeects.

1 2 3 4 5

8. Sometimes [your patient] does not pay full attention to what you are trying to tell [him/her]

1 2 3 4 5

9. You have no worries about putting your reputation in [your patient]'s hands e.g. as part of a 360 degree review, where patients rate their clinicians.

1 2 3 4 5

10. All in all, you have complete trust in [your patient].

1 2 3 4 5
