# Supplementary material for: Working alliance, interpersonal trust and perceived coercion in mental health review hearings
Source: Int J Ment Health Syst. 2011 Nov 10;5:29. doi: 10.1186/1752-4458-5-29 (PMC3227564; doi:10.1186/1752-4458-5-29)
Supplement: Additional file 2 — MacArthur Scales for Perceived Coercion, Procedural Justice and Impact of Hearing adapted for mental health hearings [2,3]. [file 1752-4458-5-29-S2.DOC]

# Additional files

### Additional file 2 – MacArthur Scales for Perceived Coercion, Procedural Justice and Impact of Hearing adapted for mental health hearings [2,3].

**Patient’s version**

**Modified MacArthur Perceived Coercion Scale (MPCS)**

Date______________ Patient’s code_____________________

| How much freedom did you have to agree or disagree with the decision of the Tribunal / Review Board? e.g. I felt free to agree or disagree with the decision of the Tribunal/Review Board.  I mostly didn’t feel free I mostly felt free  1 2 3 4 5 6 7 |
| --- |
| Did you have choices concerning the decision of the Tribunal / Board e.g. I chose to stay or leave the hospital.  I had no choices I definitely had choices  1 2 3 4 5 6 7 |
| Whose idea (initiative) was it to hold the Tribunal / Board? e.g. It was my idea to go before the Tribunal / Board.  Someone else’s initiative Mostly my initiative  1 2 3 4 5 6 7 |
| How much control did you have over the decision? e.g. I had a lot of control over the decision of the Tribunal / Board, whether I stayed or left the hospital.  Mostly others had control Mostly I had control  1 2 3 4 5 6 7 |
| How much influence did you have over the decision? e.g. I had more influence than anyone else over the decision of the Tribunal / Board, whether I stayed or left the hospital.  Mostly what other people wanted Mostly what I wanted  1 2 3 4 5 6 7 |

**Patient’s version**

**Perceived Procedural Justice (legal)**

**Date______________ Patient’s code_____________________**

Not at all = 1, definitely = 7

Voice: I had an opportunity to tell the Tribunal / Board and my legal advocate information about my personal and legal / health situation

Not at all Definitely

1 2 3 4 5 6 7

Interest: The Tribunal / Board and my legal advocate seemed genuinely interested in me as a person

Not at all Definitely

1 2 3 4 5 6 7

Respect: The Tribunal / Board and my legal advocate treated me with respect

Not at all Definitely

1 2 3 4 5 6 7

Fairness: The Tribunal / Board and my legal advocate treated me fairly

Not at all Definitely

1 2 3 4 5 6 7

Satisfaction: I was satisfied with how The Tribunal / Board and my legal advocate treated me and dealt with my case

Not at all Definitely

1 2 3 4 5 6 7

“Are you satisfied with the decisions made about your case today?”

Not at all Definitely

1 2 3 4 5 6 7

**Patient’s version**

**Perceived Procedural Justice (re psychiatrist)**

**Date________________________ Patient’s code____________________**

Not at all = 1, definitely = 7

Voice: I had an opportunity to tell my consultant psychiatrist information about my personal and legal / health situation

Not at all Definitely

1 2 3 4 5 6 7

Interest: My consultant psychiatrist seemed genuinely interested in me as a person

Not at all Definitely

1 2 3 4 5 6 7

Respect: My consultant psychiatrist treated me with respect

Not at all Definitely

1 2 3 4 5 6 7

Fairness: My consultant psychiatrist treated me fairly

Not at all Definitely

1 2 3 4 5 6 7

Satisfaction: I was satisfied with how my consultant psychiatrist treated me and dealt with my case

Not at all Definitely

1 2 3 4 5 6 7

“Are you satisfied with the decisions made about your case today?”

Not at all Definitely

1 2 3 4 5 6 7

**Patient’s version**

**Impact of Hearing**

**Date____________________________Patient’s code_________________**

As a result of (being in court, at Mental health Review Board, Mental Health Tribunal) and compared with how you felt prior to that, do you feel

Worse Better

1 2 3 4 5 6 7

Upset Calm

1 2 3 4 5 6 7

Less respected More respected

1 2 3 4 5 6 7

Confused Informed

1 2 3 4 5 6 7

Less hopeful More hopeful

1 2 3 4 5 6 7

Good Bad (globally, over all)

1 2 3 4 5 6 7

NG Poythress, Petrila J, McGaha A, Boothroyd R **Perceived coercion and procedural justice in the Broward mental health court**, *Int J Law Psychiatry* 2002, **25**, 517-533.

**Clinician’s version**

**Modified MacArthur Perceived Coercion Scale (MPCS)**

**Date____________________ Clinician’s code_________________________**

| How much freedom did you have to agree or disagree with the outcome? e.g. I felt free to agree or disagree with the outcome of Tribunal/Review Board.  I mostly didn’t feel free I mostly felt free  1 2 3 4 5 6 7 |
| --- |
| How much choice did you have concerning the outcome? e.g. I chose for the patient to stay or leave the hospital.  I had no choices I definitely had choices  1 2 3 4 5 6 7 |
| Whose idea (initiative) was it to go before the Tribunal / Board? e.g. It was my idea for the patient to go before the Tribunal / Board.  Someone else’s initiative Mostly my initiative  1 2 3 4 5 6 7 |
| How much control did you have over the outcome? e.g. I had a lot of control over the outcome of the Tribunal / Board, whether the patient stayed or left the hospital.  Mostly others had control Mostly I had control  1 2 3 4 5 6 7 |
| How much influence did you have over the outcome?  e.g. I had more influence than anyone else over the decision of the Tribunal / Board.  Mostly what other people wanted Mostly what I wanted  1 2 3 4 5 6 7 |

**Clinician’s version**

**Perceived Procedural Justice (re lawyers)**

**Date____________________ Clinician’s code_________________________**

Not at all = 1, definitely = 7

Voice: I had an opportunity to tell the Tribunal / Board and patient’s legal advocate information about my patient’s legal / health situation

Not at all Definitely

1 2 3 4 5 6 7

Interest: The Tribunal / Board and patient’s legal advocate seemed genuinely interested in me as a professional

Not at all Definitely

1 2 3 4 5 6 7

Respect: The Tribunal / Board and patient’s legal representative treated me with respect

Not at all Definitely

1 2 3 4 5 6 7

Fairness: The Tribunal / Board and patient’s legal representative treated me fairly

Not at all Definitely

1 2 3 4 5 6 7

Satisfaction: I was satisfied with how the Tribunal / Board and patient’s legal advocate treated me and dealt with my case

Not at all Definitely

1 2 3 4 5 6 7

“Are you satisfied with the decisions made about your case today?”

Not at all Definitely

1 2 3 4 5 6 7

**Clinician’s version**

**Perceived Procedural Justice (re patient)**

**Date____________________ Clinician’s code_________________________**

Not at all = 1, definitely = 7

Voice: I had an opportunity to tell my patient information about his/her personal and legal / health situation

Not at all Definitely

1 2 3 4 5 6 7

Interest: My patient seemed genuinely interested in my professional opinion

Not at all Definitely

1 2 3 4 5 6 7

Respect: My patient treated me with respect

Not at all Definitely

1 2 3 4 5 6 7

Fairness: My patient treated me fairly

Not at all Definitely

1 2 3 4 5 6 7

Satisfaction: I was satisfied with how my patient treated me and dealt with his/her case

Not at all Definitely

1 2 3 4 5 6 7

“Are you satisfied with the decisions made about your patient’s case today?”

Not at all Definitely

1 2 3 4 5 6 7

**Clinician’s version**

**Impact of Hearing**

**Date____________________ Clinician’s code_________________________**

As a result of (being in court, at Mental health Review Board, Mental Health Tribunal) and compared with how you felt prior to that, do you feel

Worse Better

1 2 3 4 5 6 7

Upset Calm

1 2 3 4 5 6 7

Less respected More respected

1 2 3 4 5 6 7

Confused Informed

1 2 3 4 5 6 7

Less hopeful More hopeful

1 2 3 4 5 6 7

Good Bad (globally, over all)

1 2 3 4 5 6 7
